# Supplementary material for: Analysis of a self-supporting shell concrete roof with nonlinear coupled evolutive material parameters
Source: PLoS One. 2025 Jul 21;20(7):e0325856. doi: 10.1371/journal.pone.0325856 (PMC12279159; doi:10.1371/journal.pone.0325856)
Supplement: Attachment — Submitted filename: 3-Review on Self-supporting shell-final.pdf [file pone.0325856.s004.pdf]

Review

PONE-D-24-21940

ANALYSIS OF A SELF-SUPPORTING SHELL CONCRETE ROOF WITH NONLINEAR COUPLED EVOLUTIVE MATERIAL PARAMETERS.

PLOS ONE

This paper considers the thermochemical and hydro-mechanical (TCHM) behavior of Concrete in the analysis. Overall, this is a fascinating topic. My main concern is that it is unclear how many specimens were used in the experimental test to determine the concrete characteristics over time (shrinkage, deflection, thickness). It is not proper to conclude based on the few specimens. This is an intriguing topic, and the overall approach is valid; I suggest it be considered for publishing after careful language editing and resolving the issues mentioned below.

Area to improve

Major:

- 1.1. **It is not clear how many specimens were used in the experimental test to determine the concrete characteristics over time (shrinkage, deflection, thickness). It is not true to conclude based on the few specimens.**
- 1.2. Determine how many cubic specimens were used to determine the compressive strength of concrete. I suggest a table or chart be presented to determine each specimen's strength and how its value is related to the strength of a 'standard specimen' (cylindrical specimens) according to the code (e.g., ACI).
- 1.3. This section (4.2.2.) requires more clarification; probably, adding some more figures and diagrams will probably help.
- 1.4. The details of the FEA model are not provided.
- 1.5. The accuracy of the finite element model should be evaluated and then applied to this specific case study.

Minor:

2. Quality of writing
    - 2.1. This manuscript requires significant language editing. There are many broken sentences. It makes it hard for the reader to understand it because of the poor writing; however, this is an interesting topic, and I would suggest it for publication after careful language editing.
    - 2.2. I suggest the authors names be mentioned in the text, which increases the readability. For example: instead of Later, [7] proposed … Later, Borack [7] proposed.
    - 2.3. Passive language is more effective and common in scientific papers; please revise it carefully. For example, in the abstract, we showed… change to: results indicated that… Or on page 4, We begin by describing…

3. Novelty, problem statement
    3.1. Well identified
    3.2. The research question is clearly stated
    3.3. Essential literature was mentioned; however, the references are not very recent. More recent works should be considered (2022-25).
4. Title, Abstract, keywords
    4.1. Add more general and specific keywords to help your work be identified more easily by potential readers (e.g., some keywords for containing shell structures, NONLINEAR COUPLED EVOLUTIVE MATERIAL, and thermochemical and hydro-mechanical (TCHM) behavior of concrete)
    4.2. Passive language is more effective and common in scientific papers; please revise it carefully. For example, in the abstract, we showed… change to: results indicated that…
    4.3. The abstract doesn't cover the method explicitly used. Please add a few sentences. Additionally, the experimental test to provide the data should be included.
5. Introduction
    5.1. Define very briefly (2-3 sentences) what 'NONLINEAR COUPLED EVOLUTIVE MATERIAL' means in this study in the introduction.
6. Method
    6.1. The equation provided on page 4, line 126, doesn't have a number. Additionally, provide additional information on how (b) is determined in this formulation.
    6.2. Page 7, line 191: Recommended value of k = 0.7; this should be backed by a reference.
    6.3. In many cases, there is a lack of information/description of the variables and constants in the formulas. (e.g., Eq19, Eq37, Eq 24, …).
7. Results
    7.1. Add some description (maximum a paragraph) in the manuscript on 'Calculation algorithm implementing the proposed model' presented in Figure 2. It is not clear what happens in this algorithm. The left part (read connection to the left line) appears to be redundant (?).
    7.2. Suggestion: A comparison of this shell while designed with conventional methods and when these defects are considered will be beneficial and add more weight to your work. (optional)
8. Conclusion
    8.1. The conclusion requires more specific findings. It is too short and doesn't contain all the findings. The main findings and conclusions and probable impact of the work could be mentioned here.
9. Figures, Tables, charts
    9.1. In Table 1, determine if the water is in liters or kg.?
10. Rewrite the author's contribution section by determining who has done what part of the work in detail. Please refer to the journal guidelines. (optional)
11. Most references are outdated; please mention and relate some recent publications. (2023-25)
