# Supplementary material for: Analysis of a self-supporting shell concrete roof with nonlinear coupled evolutive material parameters
Source: PLoS One. 2025 Jul 21;20(7):e0325856. doi: 10.1371/journal.pone.0325856 (PMC12279159; doi:10.1371/journal.pone.0325856)
Supplement: Attachment — Submitted filename: Reponse to Reviewers-1.docx [file pone.0325856.s002.docx]

PONE-D-24-21940

ANALYSIS OF A SELF-SUPPORTING SHELL CONCRETE ROOF WITH NONLINEAR COUPLED EVOLUTIVE MATERIAL PARAMETERS.

PLOS ONE

5. Review Comments to the Author

Please use the space provided to explain your answers to the questions above. You may also include additional comments for the author, including concerns about dual publication, research ethics, or publication ethics. (Please upload your review as an attachment if it exceeds 20,000 characters)

Reviewer #1: • Overall Strengths: The manuscript offers a novel contribution to the field by integrating multiple phenomena that affect concrete performance over time, making it particularly valuable for long-term structural design applications. The proposed TCHM model is robust and well-supported by experimental data.
• Areas for Improvement:
1. Data Interpretation: Provide more detailed explanations for intermediate results, particularly regarding the effects of hydration and delayed deformations on the overall structural behavior.

Answer : Details provided see lines 316-317 and 341-343

2. Statistical Analysis: Consider performing sensitivity analysis to further validate your model and provide deeper insights into the impact of key parameters.

Answer : Please see table 3 and lines 409-413

3. Data Sharing: Ensure that all underlying data, including raw measurements, are made fully available in accordance with PLOS ONE's data policy.

Answer : The data are available

4. Language: Simplify the presentation of some technical content to improve readability and correct minor typographical and grammatical errors.

Answer : A proofreading was done and the typos corrected.

Conclusion: The manuscript is scientifically sound and offers important insights for concrete shell design. With minor revisions to improve data accessibility, statistical rigor, and clarity, it is suitable for publication.

Reviewer #2: The paper covers an important topic, but the authors need to strengthen the logical reasoning and clarity of the results. Specifically:
1. Revise the abstract to include details about the experimental work, outcomes, and recommendations.

Answer : Details provided see lines 18-22

2. Provide more in-depth explanations in the section discussing linear shrinkage.

Answer : Details provided see lines 365-368

3. Add a paragraph outlining potential future research directions based on the current study.

Answer : Details provided see lines 447-451

4. Include a paragraph at the end of the introduction emphasizing the research significance of the study.

Answer : Details provided see lines 109-116

5. Update the references with more recent research, as there are no citations from 2024 and only two from 2023, to better support the ideas and highlight the novelty of the work.

Answer : See [13], [22] and [40].

Reviewer #3: This article aims to describe a more complete framework by which to evaluate structural deformation in thin-shelled concrete dome structures: by including the contributions of thermochemical and hydromechanical behavior (e.g. creep) of concrete, in addition to typical elastic deformation. The paper then showcases the results of the proposed methodology by an analysis of a structure designed like an aircraft, particularly in the fuselage-like portion.

While the proposed article has potential, and appears to investigate an area of interest, the article lacks clarity in a number of areas. This makes the article difficult to evaluate from the perspective of novelty, completeness, and value of the proposed method. Particularly, the article needs to better address the following areas:
1. Many of the terms that are used are not defined, and not motivated. Particularly,
a. It is not clear why the Kronecker delta is only define don the midsurface.

Answer : corrected

b. What is \mu^\rho_\alpha, which is on the last line of the first page of the document? What does it physically correspond to?

Answer : $\mu_{\alpha}^{\rho}=\delta_{\alpha}^{\rho}-zB_{\alpha}^{\rho}$

It’s the matrix which makes it possible to connect the covariant base of the 3D shell to the covariant base on the mid-surface.

c. Does the document use summation notation on indices or not?

Answer : In some relationship yes not in others.

d. What is the physical meaning of \psi (the first equation of the second page?

Answer : rate of change of volume. Addition added see line 33.

e. When \omega_L is introduced on page 5, it should be explicitly mentioned (using the language from the start of the text) that this is the free water content.

Answer : corrected

f. A description of the variables and parameters in Equations 2 and 3 are not present in the text. What are these? Without physical intuition and meaning, it is hard to motivate why the method of choice by the authors is appropriate.

Answer : description done see lines 147-152

g. What does “The drying parameter A and \delta are numerically adjusted after the resolution” mean (see line 141 of the text)? Are these parameters that are fit based on experimental data? Or to match a given deformation? Please clarify.

Answer : The clarification provided see lines 155-156

h. Is k of line 148 the same as \kappa in line 147? What do these calibration parameters correspond to? What is their physical meaning?

Answer : input error correction provided see lines 161-163

i. Variables in Section 3.3 lack definition, explanation, and intuition. Please label these variables and describe what they mean. What is the intuition that a practitioner can use to understand these? Also, for equation 13, what is the meaning of the numbers 1000 for concretes with silica fume and 3200 for those without silica fume? What is the repercussion for changing these numbers in this way? Where did these numbers come from?

Answer : details provided in section 3

j. Should Ac and Bc of equations 179 and 180 be A_c and B_c, where the _ denotes a subscript?

Answer : The clarification provided see lines 193-198

k. The right hand side of the equation r = … on line 181 is not legible

Answer : modification provided see line 199

l. Variables in Section 3.4 also lack definitions and any understanding. As a result, it is hard to evaluate the merit of the proposed method or determine what the proposed operations are doing.

Answer : comments provided see lines 191-193 and 204-209

m. The sentences between lines 187 and 188 are not clear.

Answer : modification provided see line 205
n. The variables in Section 3.5 also lack definitions and any sense of intuition

Answer : clarification provided

o. Is U_{ad}, on line 197, the Cartesian product of Sobolev spaces? Or are the x variables in line 198 actually x’s. Please clarify.

Answer : clarification provided, see also line 224

p. It would be nice to see on or near line 201 that this is essentially the equations of linear elasticity and lambda and mu are Lame parameters.

Answer : clarification provided

q. Again, Equations 24 through 27 are not readable and lack any intuition. Where are these coming from? What do they mean? Each in isolation can be digested, but when all of them are crowded into a few very small lines, they remain indecipherable.

Answer : clarification provided

r. What are the variables used in Equations 28 and 29? Only some of them are declared in the subsequent lines. What about alpha? \bar{v} (Poisson’s ratio?) Please clarify. It is also hard to see which parentheses lead to multiplications and which ones are function evaluations. Could you use a dot product dot to indicate multiplication?

Answer : clarification provided

s. Lines 218 through 248 again lack any intuition. From what equations are these equations substituted into? Again, a lack of understanding, intuition, and definitions on most of the variables used herein make this feel more like spewing a list of equations rather than doing anything to build intuition, motivation, or confidence in the methods used.

Answer : After the variational formulation we reorganise the equation by grouping similar terms in order to facilitate numerical implementation in the FEM code.

t. What kinds of isoparametric Lagrangian symplectic triangle finite elements are used? Quadratic?

Answer :Only Triangle elements	are used in the mesh with Quadratic polynomials as shape functions for tranverse displacements and linear for the others

u. It appears that some variables may be overloaded (i.e. used twice to define different phenomena), such as A in line 141 and in line 179. These overloads should be removed---particularly because there is already a significant amount of different information being conveyed.

Answer : correction provided

v. The Gauss tensor, Q_{\alpha \beta}, appears to be first introduced in line 375 of the document. Please introduce earlier and define earlier.

Answer : correction provided see lines 279-280

2. Some of the symbols defined appear to never be used, such as the Christoffel symbol. Please review the article to better clarify symbols used and to remove any extraneous symbols.

Answer : Christoffel symbol is used see line 277

3. The article currently feels like a compilation of many different methods into a single method. However, the choice of these methods is not motivated. Do the assumptions of these methods work in conjunction with each other? Why, among all methods, were these methods chosen? Is there novelty beyond simply putting everything together? If so, what is it?

Answer : It’s not a compilation of methods . Unlike in earlier works in this field where the different phenomenon were considered seperately. In reality they are concomitant and should be considered dependent in a realistic analysis. Herein, the complex coupled equations are considered and numerical calculations have been successfully implemented on the model. Figure 2 presents the skeleton of the proposed model.

4. Why are different specimen dimensions used for shrinkage and drying experiments in Section 4.2.2? Were both following a methodology published in the literature? If not, why the variation?

Answer : Yes both dimensions were used in some publications in the litterature

5. What variables are fit in lines 301 and 302? Are these corresponding to Equations 3 and 5? If so please clarify.

Answer : clarification provided

6. How was the time evolution of water content measured in Section 5.1.ii? Please also clarify how measurements were taken and the meaning of the quantities in the legend of Figure 5.

Answer : clarification provided see lines 359-362

7. How were damage evolution parameters determined? Are they based on experimental data, or on numerical simulations, based on reference from other publications?

Answer : clarification provided, based on references.

8. There is not a clear distinction between what measurements and data relate to experimental data and what relates to numerical data throughout Section 5.2. Please clarify.

Answer : In section 5.2 this concerns the presentation of the simulation results, recalling the useful data where needed.

9. Many of the figures show behavior that has not leveled off asymptotically, such as in Figure 12, Figure 13, and Figures 14. These are for structural information that should stabilize. Why is such a stabilization not present? Is this due to lack of computing resources, or ability to measure? I question the validity of the model if, for example, the thickness of the shell linearly deteriorates indefinitely, or if the strain at node E continues to change indefinitely.

Answer : we observe quasi-asymptotic behavior, The simulations are carried out over 50 days. We remain convinced based on the trends that asymptotic behavior will occur. The available resources took us 8 days for a compilation with this spatial and temporal mesh step.

10. Some of the labels and graphics are difficult to read because lines are present on top of other lines. If this is the case, could the authors please put nodes that correspond to different graph lines in different locations so figures are more easily decipherable?

Answer : clarifications provided.

11. It is not clear if there was any validation of the model on the actual constructed structure. If not, please clarify why. For such a complex model, there should be some validation that the results produced are meaningful and give higher-fidelity results than significantly simpler models.

Answer : Yes, on the basis of project data (architectural design, concrete mixture and climatlc conditions) we reproduced the experiment to carry out the various calibrations. The finite element validation was tested on several structures (real and benchmark in the literature [40-41])

In addition, there are a number of typographical errors throughout the article (e.g. Finite Element Method should all be lower-case in the abstract, ThermoChemical and HydroMechanical should also be lower-case and hyphenated, the mapping u from the closure of the domain should probably go to R^3, etc). Overall, this did not impede my ability to read and follow the article, but removing the typos would be important prior to publication. Also, why is the Reissner-Mindlin shell element referred to as Q4GG? If this is particular notation to some software, please reference it or remove it. Otherwise, please cite the specific version of the Reissner-Mindlin shell that is the Q4GG element used.

Answer : clarifications provided.

Finally, some of the superscripts and subscripts on symbols---particularly those using a percent sign as a subscript----were difficult to read and should be typeset in a more readable manner.

Answer : clarifications provided.
