# Supplementary material for: Analysis of a self-supporting shell concrete roof with nonlinear coupled evolutive material parameters
Source: PLoS One. 2025 Jul 21;20(7):e0325856. doi: 10.1371/journal.pone.0325856 (PMC12279159; doi:10.1371/journal.pone.0325856)
Supplement: Attachment — Submitted filename: comments authors_2.docx [file pone.0325856.s005.docx]

Review

PONE-D-24-21940 ANALYSIS OF A SELF-SUPPORTING SHELL CONCRETE ROOF WITH NONLINEAR COUPLED EVOLUTIVE MATERIAL PARAMETERS. PLOS ONE

The quality of the manuscript has improved significantly with the most recent revision. Many of the ideas that were unclear are now clear. However, there are still a number of remaining concerns.
1. Please make \mu^\rho_\alpha into its own equation shortly before or after line 32.

Answer : done
2. There is a host of variables that are used herein. However, the nomenclature section only contains about ½ of them. Please place all variables in the nomenclature section and define them therein so that readers know at least one place they can go to follow what is being written. Current in-text definitions are also helpful, but are insufficient (as discussed more below).

Answer : It was pruned
3. From what I can see, summation notation is used throughout. Please indicate this near the beginning of the document. If there are instances where summation notation is not used, please explicitly call these out.

Answer : Specified note
4. Please clarify on Equation 12 if \epsilon_{cd} is a computed quantity or if it is provided from experiments. If it is computed (and leads to total strain), consider moving it to the left hand side of an equation.

Answer : is a computed quantity,

5. The role of creep (Section 3.3) in the overall model. \epsilon^{cr} is defined in the beginning and used in Figure 2, but it does not seem to be present in Section 3.3. Additionally, the total creep function (Equation 14) is confusing in the following ways
a. The expression J(t,t_0) does not appear to be used anywhere else in the document. As a consequence, it is not clear how this contributes to creep (e.g. in the algorithm of Figure 2). Please clarify. How is this related to \epsilon^{cr}?

Answer : clarification given
b. The quantities E_c, E_{c28}, and s in Equation 14 are not defined.

Answer : clarification given
c. Both the self-creep and the desiccation creep are defined (in Equations 9 and 12, respectively) prior to their use in Equation 14. However, the other term (1/E_c(t_0)) is never discussed prior to its use in Equation 14. What does this represent?

Answer : clarification given
d. Why are the creep terms divided by the E_{c28} terms?

Answer : relatiobship reported in [38]
6. Quantities in Section 3.4 are undefined and often not motivated.
a. Particularly, what do Y_0, Y, A, B, \epsilon_do \epsilon, A_t, j, k, r, A_c, B, B_t, B_c, \tilde{sigma_i}, \epsilon_{eq}^{\corr}, <__>_{-}, <__>_{+}, and \gamma physically mean?

Answer : clarification made in 3.4
b. Where does someone trying to reproduce this work get information about how to get these variables? A couple are discussed as equations (e.g. Y_0), but others (e.g. k) are not. Please be clearer. The final paragraph of Section 3.4 is a good start, but is insufficient.

Answer : we have enriched and the computed values are specified in section 5.2.1
7. Quantities in Section 3.5 also need clarification.
a. Is \bar{q} a number? If so, why does it does it have superscripts in line 282? If not, why is the set of admissible displacements only the Cartesian product of 4 Sobolev spaces (i.e. 3 for u and 1 for q)?

Answer : clarification provide between lines 245-250
b. Please describe what the stretching displacement does in words.

Answer : stretching displacement allows the calculation of shear strain and stress.
c. S (in H^1(S)) is not defined. Is this the surface? The midsurface? The entire domain? It appears to be defined as the midsurface in Line 232, but it is first used in Line 224. This is confusing.

Answer : clarification provide : S is the midsurface see line 257
d. G^{\alpha \beta} (and similar indexed terms on e.g. Line 229) are never defined, though it is presumed that they relate to the contravariant bases of the shell. Are they inner products of these bases? Or subscripts of the bases? Please clarify.

Answer : clarification made  : see line 261
e. A in Line 229 is not defined, and its relationship with C in Line 234 is also unclear. Please clarify.

Answer : clarification made  : see line 259

f. Subscript indices on \epsilon^\eta and \epsilon^q in line 238 appear to be missing.

Answer : it’s correct , this is another notation

g. Tensors e, k, and Q are used in Equation 26 (Line 241), but are undefined. They appear to be defined on Lines 275, 277, and 278. Please define these shortly after their first use, rather than defining them over an entire page later.

Answer : clarification made between lines 271-277

h. The tensor Y on line 260 is not defined. Please clarify.

Answer : correction made see line 297
i. What are the terms in the linear form (i.e. L(v,\bar{y}) on line 282? Similarly, what are the terms in the integrands of Line 283. Define all terms so that the information can be used and so that it is appropriately motivated. Define them both using equations (where relevant) and verbally. If it is a linear form, why is q (an unknown as per Line 218) present in it?

Answer : clarification made
j. Notation with \partial_\alpha (e.g. line 261) and with commas in index notation (e.g. line 276) are both used. Please clarify if both are partial derivatives or if they indicate different behavior.

Answer : clarification made see line 306

8. Figure 2 is the only way to understand how all of the information from Section 3 is integrated. However, it is not well introduced in the text of Section 4. Please spend more time discussing this figure so that readers can understand how the information from Section 3 can be used in your model. Also, there appear to be many, many more input parameters for the algorithm than just the time of simulation and the number of time increments (e.g. Lame parameters and/or Young’s modulus and Poisson’s ratio, j=0.7, etc).

Answer : details provided
9. Please improve Figure 2 to
a. Include arrows between every box/shape.

Answer : The arrows have been added
b. Have a caption that more fully describes the algorithm.

Answer : done
c. If the numerical experiment is only a maximum of 50 days, why is the creep strain ignored until after 28 days? Please clarify in the text.

Answer : clarification provided
d. It appears that the algorithm is nonlinear. However, the nonlinearity is not clearly indicated (through e.g. arrows) in Figure 2. Please clarify the iterative/nonlinear process for each time step, or please clarify that the method is not linear.

Answer : clarification provided
10. The numerical implementation section spends a significant amount of discussion on how parameters were extracted using experiments. Please separate the experimental/material parameter discussion into a separate section before the numerical implementation. This will also clarify what experimental work is done and what is actually numerical.

Answer : It wil be difficult for us to separate the results because it involves both experimental and numerical work. The experimental results allow us to calibrate the parameters for the proposed model. The results were presented in order.
11. Similarly, the results and discussions section makes it unclear what results are numerical and what results are due to the model. Please explicitly declare what results are experimental and which ones are numerical. It is my understanding that almost all results are numerical except for those in the “Intermediate Results” section. Is this correct?

Answer : clarification provided see lines 415-416
12. The text between Lines 422 and 427 confuses me. Does this indicate that for a static (elastic) analysis to produce the same amount of displacement as this model (which includes creep, damage, thermal effects, etc), the static analysis would have to apply a force 183x larger than that applied when including creep, damage, etc?

Answer : we are trying to show that nor taking into account the delayed effects, therefore the specificities of concrete, in the calculation phase would be a dangerous decision.
13. Temperatures on each day are shown in Figure 8. However, analysis was only performed for 50 days. On what day did this analysis begin? Which temperature values were used, and over which of 50 days of the 365 displayed?

Answer : we use 50 days, see lines 448-449.
14. What time steps were used for this analysis? One time step per day? What kind of solver was used? Implicit or explicit?

Answer : see lines 458-459.
15. Please clarify in the text why you can be confident that the chosen time step increments were appropriate and stable. Similarly, if there is a nonlinearity in each time step, please indicate why you can have confidence that the number of nonlinear iterations was appropriate to resolve behavior in each time step.

Answer : We did not use a resolution scheme for time discretization, we directly calculate the value at a given time. For each step we use the algorithm in figure 2
16. The authors indicate that they believe that their simulations approach asymptotic behavior. However, Figures 12, 13, and 14 do not seem to demonstrate that the model reaches an asymptotic stability over the course of 50 days. In the rebuttal, authors have declared that they lack computational ability to perform additional analysis and that this one already took 8 days. This makes sense. However, the authors should clarify in the conclusions that asymptotic behavior for their model is believed, but that further research is needed to clarify that the proposed method is applicable for long timeframes. Particularly, it is not clear to the reviewer that displacement will not increase indefinitely and that the model accurately represents what would be anticipated experimentally. This deficiency does not discredit this work; however, this lack of clarity should be openly stated.

Answer : In all the documents consulted, therefore some in reference which deal with a similar theme have an asymptotic behavior , also our results show us a creep dominance therefore the curve will be a asymptotic given the shape of the delay function.
17. Readability issues:
a. Please revisit the sentence starting on line 86 for clarity.

Answer : clarification provided, see lines 90-92
b. Line 169: “Given the small size of the shell…” Should this be “Given the small thickness of the shell…”?

Answer : corrected, see 172
c. The sentence beginning on line 190 is incomplete.

Answer : We have completed see lines 203-207
d. Please indicate units of liters using a capital L rather than a lower-case L.

Answer : correction provided

Reviewer #4: This paper considers the thermochemical and hydro-mechanical (TCHM) behavior of
Concrete in the analysis. Overall, this is a fascinating topic. My main concern is that it is unclear how many specimens were used in the experimental test to determine the concrete characteristics over time (shrinkage, deflection, thickness). It is not proper to conclude based on the few specimens.

This is an intriguing topic, and the overall approach is valid; I suggest it be considered for publishing after careful language editing and resolving the issues mentioned in the attachment.

Area to improve

Major:

1.1. It is not clear how many specimens were used in the experimental test to determine the concrete characteristics over time (shrinkage, deflection, thickness). It is not true to conclude based on the few specimens.

Answer : we have used 12 specimens in total

1.2. Determine how many cubic specimens were used to determine the compressive strength of concrete. I suggest a table or chart be presented to determine each specimen's strength and how its value is related to the strength of a ‘standard specimen’ (cylindrical specimens) according to the code (e.g., ACI).

Answer : Three test specimens were removed from the immersion ank on the 28th day and sent to an approved laboratory. This laboratory carried out the crushing in accordance with the NF P 18-406 standard prescribed by us.

1.3. This section (4.2.2.) requires more clarification; probably, adding some more figures and diagrams will probably help.

Answer : corrected

1.4. The details of the FEA model are not provided.

1.5. The accuracy of the finite element model should be evaluated and then applied to this specific case study.

Answer : it’s done in previous work [40-41]

Minor:

2. Quality of writing

2.1. This manuscript requires significant language editing. There are many broken sentences. It makes it hard for the reader to understand it because of the poor writing; however, this is an interesting topic, and I would suggest it for publication after careful language editing.

Answer : The corrections have been provided

2.2. I suggest the authors names be mentioned in the text, which increases the readability. For example: instead of Later, [7] proposed … Later, Borack [7] proposed.

Answer : done

2.3. Passive language is more effective and common in scientific papers; please revise it carefully. For example, in the abstract, we showed… change to: results indicated that… Or on page 4, We begin by describing…

Answer : revisions have been done

3. Novelty, problem statement

3.1. Well identified

3.2. The research question is clearly stated

3.3. Essential literature was mentioned; however, the references are not very recent. More recent works should be considered (2022-25).

4. Title, Abstract, keywords

4.1. Add more general and specific keywords to help your work be identified more easily by potential readers (e.g., some keywords for containing shell structures, NONLINEAR COUPLED EVOLUTIVE MATERIAL, and thermochemical and hydro-mechanical (TCHM) behavior of concrete)

Answer : Keywords added

4.2. Passive language is more effective and common in scientific papers; please revise it carefully. For example, in the abstract, we showed… change to: results indicated that…

Answer : corrections provided

4.3. The abstract doesn’t cover the method explicitly used. Please add a few sentences. Additionally, the experimental test to provide the data should be included.

Answer : a few sentences have been added

5. Introduction

5.1. Define very briefly (2-3 sentences) what ‘NONLINEAR COUPLED EVOLUTIVE MATERIAL’ means in this study in the introduction.

Answer : Firstly, we have internal dissipation, which is linked to phenomena such as shrinkage, creep, etc. These independent specificities will give concrete non-linear behavior. We will therefore have material or rheological non-linearity.

6. Method

6.1. The equation provided on page 4, line 126, doesn’t have a number. Additionally, provide additional information on how (b) is determined in this formulation.

Answer : Corrections made

6.2. Page 7, line 191: Recommended value of k = 0.7; this should be backed by a reference.

Answer : Correction made

6.3. In many cases, there is a lack of information/description of the variables and constants in the formulas. (e.g., Eq19, Eq37, Eq 24, …).

Answer : Corrections made

7. Results

7.1. Add some description (maximum a paragraph) in the manuscript on ‘Calculation algorithm implementing the proposed model’ presented in Figure 2. It is not clear what happens in this algorithm. The left part (read connection to the left line) appears to be redundant (?).

Answer : Clarification provided

7.2. Suggestion: A comparison of this shell while designed with conventional methods and when these defects are considered will be beneficial and add more weight to your work. (optional)

Answer : We present a comparison of lines 502-507

8. Conclusion

8.1. The conclusion requires more specific findings. It is too short and doesn’t contain all the findings. The main findings and conclusions and probable impact of the work could be mentioned here.

Answer : The conclusion was added

9. Figures, Tables, charts

9.1. In Table 1, determine if the water is in liters or kg?

Answer : correction done

10. Rewrite the author's contribution section by determining who has done what part of the work in detail. Please refer to the journal guidelines. (optional)

Answer : added

11. Most references are outdated; please mention and relate some recent publications. (2023-25

Answer : see [22], [40],
